# Nickel-copper-cobalt mixed oxide electrode material for high performance asymmetric supercapacitor

**DOI:** 10.1038/s41598-024-61625-y

**Published:** 2024-05-11

**Authors:** M. Manikandan, E. Manikandan, V. Swetha, S. Kurpaa, Sukkrishvar Vijay, V. Kiruthika

**Affiliations:** 1 Centre for Innovation and Product Development, Vellore Institute of Technology, Chennai, 600127 India; 2 School of Electronics Engineering, Vellore Institute of Technology, Chennai, 600127 India

**Keywords:** Mixed metal oxides, Asymmetric supercapacitor, Energy density and power density, Chemistry, Energy science and technology, Materials science

## Abstract

Nickel copper cobalt oxide (NiCuCoO) ternary metal oxide nanoparticles were synthesized by employing the hydrothermal method. NiCuCoO electrode demonstrates a specified capacity of 596 C g^−1^ at 1 A g^−1^, high capacitance retaining of 99% even if 1000 sequences at the density of current 10 A g^−1^, and significant extended cyclic strength over 1000 sequences. The gathered asymmetric supercapacitor (ASC) tool via NiCuCoO as the cathode and activated carbon as anode materials achieve a specified capacity of 168 C g^−1^ at a current density of 1 Ag^−1^, an excellent capacity retaining of 95% even later than 5000 sequences at a density of current 10 A g^−1^. The fabricated device exhibits a high density of energy and power is 96 Wh kg^−1^ and 841 W kg^−1^. The prepared material confirms an excellent capacitance routine, so this work represents for a next-generation energy storage device.

## Introduction

The significance of energy storage is escalating as every number of renewable sources of electricity is expanding dramatically^1–3^. Technologies with high power density, energy density, along with adaptive energy storage be necessitated considering the rapid emergence of electric-powered automobiles and portable consumer electronics. The influence of electrochemical devices that store energy is growing because of their exceptional efficiency, adaptability, electrical conductivity, and utility. Batteries and supercapacitors are the most efficient players among the varied devices that store electrochemical energy (SCs). The major distinction among SCs and batteries are the charge storage process, and also the materials along with framework. SCs have the distinct advantage of bridging the energy density gap between ordinary capacitors and batteries^4–7^. Supercapacitors have exceptional cycle capacity and can operate in a broad range of temperatures without incurring markedly from capacity loss. In estimating energy storage, supercapacitors also exhibit extreme non-linear behavior, mandating the use of intricate modeling techniques^8–10^. Additionally, a hybrid storage device that enhances operational durability can be constructed by incorporating supercapacitors and rechargeable batteries^6,7,11^. Metal oxides comprises of metal ions and organic linkers held by strong chemical bonds. They have made substantial progressions in energy storage devices as they can perform as templates or precursors in the fabrication of porous, crystalline transition metal oxides such as Co_3_O_4_^12^, NiO^13^, MnO^14^, CoO^15^, and CuO^16^.

Ternary or higher order metal oxides are commonly used as positive electrodes in supercapacitors due to their high specific capacitance, composition tuning, and low cost. The rationale for studying ternary metal oxide is as follows: Mixed oxides with different valence states have higher electrochemical conductivity and specific capacitance (Cs) than monometallic and bimetallic oxides^17–20^. Because of their enhanced electrochemical activity as well as greater stability, ternary metal oxides have lately been promoted to be utilized in super capacitor applications. For example, Ni-Zn-Co^1^, Zn-Ni-Co^10^, Cu–Zn-Co^21^, Zn-Ni-Co^22^, Zn-Mn-Co^23^, has gained attention. Hu et al. enlarged flower-like Ni-Zn-Co oxide NWs among specified capacity (776 F g^−1^) also real capacity of 1.16 F cm^−2^ (2 A g^−1^), long cycle stability of 88.9% with capacity retaining of 73.8% (32 A g^−1^) with every collected aqueous symmetric supercapacitor having an energy and density of power is 44.5 Wh kg^−1^ at 880 W kg^−1^^1^. Putjuso et al. combined Co_x_Zn_1−x_Fe_2_O_4_ nanoparticles by hydrothermal method at 200 °C for 12 h. with highest particular capacities, 855.33 F g^−1^ (1 A g^−1^), by a high-quality rate ability retaining of 90.41% after 1000 sequences^3^. Kim et al. made-up electrodes with combined (La_0.8_Sr_0.2_Mn_0.5_Co_0.5_O_3−δ_) materials carrying redox reaction capacity through graphene nanosheets with great capacitance and exceptional withholding of 95% after 5000 series^4^. Zhang et al. showed a method to directly grow zinc-nickel–cobalt oxide@Ni(OH)_2_ NWs on a CNT fiber among an excellent capacity of 2847.5 F cm^−3^ (1 mA cm^−2^), a huge capacity of 94.67 F cm^−3^ amid an density of energy is 33.66 mWh cm^−3^^10^. Vijayakumar et al. used a hydrothermal method to create Cu–Zn–Co oxide NFs on Ni-foam, and FESEM revealed a thin NFs-like morphology with a most specified capacities of 215 C g^−1^ (5 mV s^−1^) and 178 C g^−1^ (1 A g^−1^)^17^. Hussain et al. The Zn-Mn-Co NNs electrode revealed a specified capacitance of 849 C g^−1^ (1 A g^−1^) after 8000 cycles, while the hybrid supercapacitor tool with Zn-Mn-Co//AC NNs electrode confirmed a high specified capacities of 158 C g^−1^ (1 A g^−1^) with a density of energy and power is 35.5 W h kg^−1^ and 7.5 kW kg^−1^ moreover 100% initial capacity retaining over 7000 sequences^19^. Huang et al.^24^ optimized a Zn-Ni-Co TOH-130 NWs electrode with an specified capacity of 305 F g^−1^ (3 mA cm^−2^) and a limited density of is energy and power 2.43 m Wh cm^−3^ and 6 m W cm^−3^. Guo et al. projected self charging electrochromic supercapacitor devices based on ESCD and sidling mode direct-current triboelectric nanogenerators^25^. The carbyne high frequency symmetric supercapacitor has high energy density of 703.25 μF V^2^ cm^−2^ at 120 Hz surpassing current commercial operations electrolytic filters and many reported alternating current line supercapacitors^26^. Choi et al. built a photopatternable solid electrolyte with a high ionic conductivity of 10 mS cm^−1^ at room temperature, as well as outstanding thermal stability and mechanical integrity^27^. Photonic bio-WORM memory and lighting devices were created to hold information and explore photovoltaic properties. Bionanoferritin cages engineered with ANADO-LUCA based ruthenium and photosensitive cross-linkers were effectively constructed^28^.

**Scheme 1:**
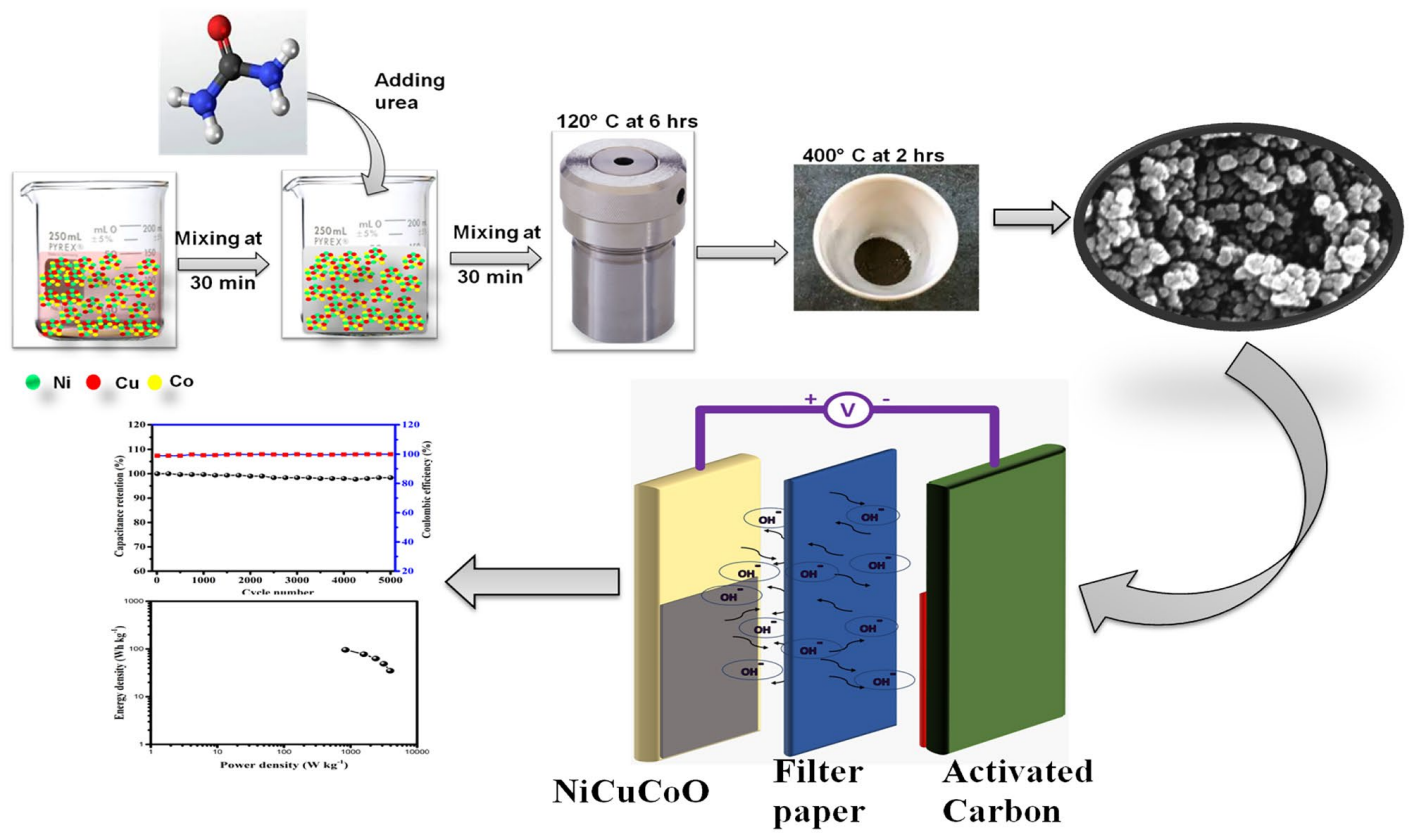
Design for the synthesis of electrode material and assembly of the ASC device.

Here we present a practical hydrothermal method to fabricate nanostructures of ternary metal oxides (NiCuCoO). Elevated specified capacities and exceptional sequencing permanence are displayed by the NiCuCoO electrode. At the same time, the NiCuCoO nanoparticles and activated carbon be worn to create the positive and negative electrodes for the ASC device. The electrode also has a high density of energy and power, next to an excellent electrochemical cohesion, showing that it could be used in energy storage. According to our information, the use of flexible mesoporous carbon cloth-supported ternary metal oxide (NiCuCoO) as an electrode for elevated concert supercapacitors is described in this report for the first time. The preparation and consecutive production of the matching ASC device are presented in Scheme 1.

## Experimental section

### Preparation of NiCuCoO nanoparticles

NiCuCoO nanoparticles were synthesized utilizing a hydrothermal method and then subjected to calcination treatment route. Typically, 60 ml of deionized water was added to dissolve 2 mmol of Cu(CH_3_COO)_2_H_2_O and Ni (CH_3_COO)_2_H_2_O, 6 mmol of Co(CH_3_COO)_2_H_2_O, 40 mmol of urea, and then the mixture was stirred magnetically for 60 min. The resulting combination is subsequently put into a stainless-steel autoclave with Teflon lining moreover maintained next to 120 °C for 6 h. Every yield is gathered, cleaned through deionized water with absolute alcohol for three times. After the end results were harden at 400 °C for two hours in an oven with an air ambiance and a warm rate of 3 °C min^−1^, the NiCuCoO nanoparticles were subsequently formed^29^.

### Materials characterization

Powder X-ray diffraction (XRD) analysis with PANalytical XRD X'pert pro and Cu K radiation (= 1.5418 A) was use to examine the formation of phase and crystal arrangement of the synthesized materials. The surface morphology and size of particles of the created materials were checked up via a scanning electron microscope (SEM) Hitachi S-3000 H. The electrochemical efficacy of the electrode materials for supercapacitor application was estimated during a workstation for electrochemical analysis (Biologic- SP300), cycles of voltammetry (CV), galvanostatic charge–discharge (GCD), and electrochemical impedance spectroscopy (EIS).

### Preparation of electrode for supercapacitor

To make every functional electrode, the active substances acetylene black and polyvinylidene fluoride (PVDF) were varied in an 80:15:5 weight ratio. To make homogeneous slurry, 0.5 ml of N-methyl-2-pyrrolidone (NMP) was use as in the black, and the mixture was constantly opinion in a mortar. The homogeneous slurry was applied to a nickel foam current collector (2 × 2 cm^2^) and dried in an air oven for 12 h at 80 °C. The mass of the dynamic substance (2 mg) was optimized by calculating the mass of the carbon cloth earlier than and later than covering. In a 3-electrode arrangement, active material-coated carbon cloth, Pt foil, and Ag/AgCl performed as the working, counter, and reference electrodes, correspondingly. The CV and GCD dimension be performed using 3 M KOH to be the electrolyte and varying sweep rates ranging as of 5 to 100 mV s^−1^ and density of current ranging as 1 to 10 A g^-1^. An alternative current (AC) through a voltage of 5 mV amplitude be applied at frequencies between 100 kHz to 10 mHz in the direction of EIS. The particular weight of the electrode substances was determined via the subsequent method^6,7^.

1 $$C=\frac{I\Delta t}{m\Delta v}.$$

### Hybrid asymmetric supercapacitor cell fabrication

The asymmetric supercapacitor (ASC) full cell was fabricated using NiCuCoO as positive electrode and activated carbon as negative electrode in a 3 M KOH electrolyte. The charge stability determined the mass percentage of the positive and negative electrodes as follows^6,7^.

2 $${q}_{+}={C}_{+}\times {m}_{+}$$

3 $${q}_{-}={C}_{-}\times {m}_{-}$$

4 $$\frac{{m}_{+}}{{m}_{-}}=\frac{{C}_{-}}{{C}_{+}},$$

where, q_+_ and q_-_ correspond to the positive and negative electrodes charges, correspondingly. The positive and negative electrode masses are represented by m + and m −, in that order. C + and C − are the specified capability of the positive (993 F g^−1^) and negative (104 F g^−1^) electrodes in the 3-electrode classification at a constant density of current 2 A g^−1^, respectively. The mass ratio between positive and negative electrodes for optimal electrochemical operation of ASC was estimated to be 0.10. The subsequent equation was worn to estimate the specified capability (Csp), coulombic efficiency (η), density of energy (E), and density of power (P) of the constructed ASC device^6,7^.

5 $$Q= \frac{I\Delta t}{M}$$

6 $$\eta = \frac{{\Delta t}_{discharge}}{{\Delta t}_{charge}}\times 100\%$$

7 $$E= 1/2 QV$$

8 $$P= \frac{E}{\Delta t}.$$

Here, *Q* is the specified capacitance of the ASC (F g^-1^), *I* is the charge–discharge current (A), Δt is the total discharge time (sec), *M* is the entire active mass of the electrode materials (g), η is the coulombic efficiency (%), E is energy density (Wh kg^−1^) and P is power density (W kg^−1^).

## Results and discussion

### Structural and morphology

**Figure 1:**
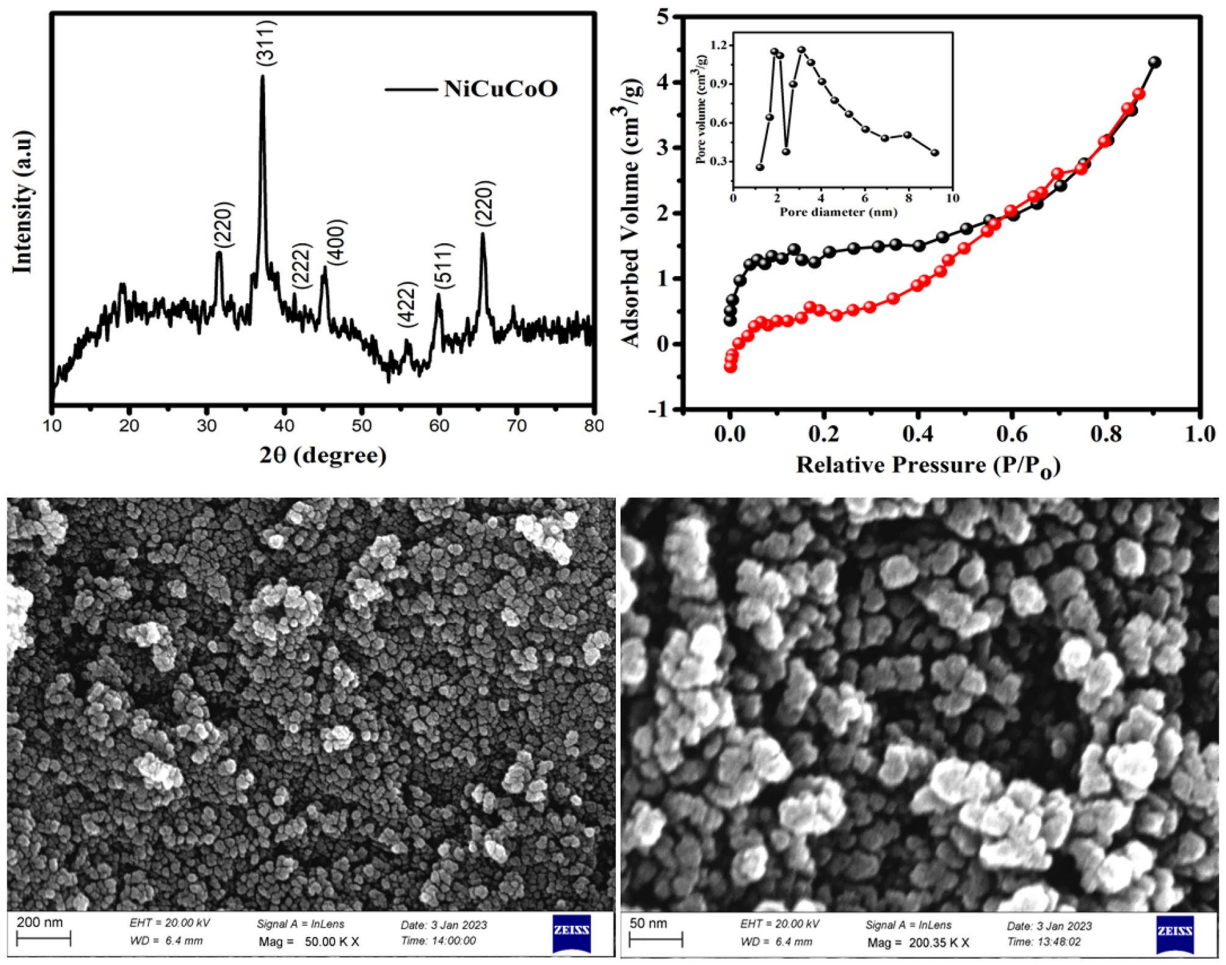
NiCuCoO NPs (**a**) XRD outline, (**b**) N_2_ adsorption/desorption isotherms (insect) pore size allocation and (**c**,**d**) FESEM image.

XRD was used to describe the crystalline structures of NiCuCoO nanoparticles, as exposed in Fig. 1a. The classic face-centered cubic symmetry phases can be seen in the XRD patterns of both CuCo_2_O_4_ NRs (JCPDS Card No. 01-1155) and NiCo_2_O_4_ NRs (JCPDS Card No. 20-0781)^29,30^, with diffraction peaks comparable to the crystalline planes of (220), (311), (222), (400), (422), (511), and (440). Except for very slight shifts, all the diffraction peaks of Ni_0.5_Cu_0.5_Co_2_O_4_ samples fit well with the standard patterns of CuCo_2_O_4_ or NiCo_2_O_4_. No other contamination diffraction peaks be showed to prove the elevated clarity of the produce. The nitrogen adsorption–desorption isotherms of NiCuCoO NPs were classify as type IV by the IUPAC are revealed in Fig. 1b. The BJH model was used to estimate the pore diameter distribution profile, which was used to further investigate the presence of mesoporous structures. NiCuCoO NPs have a BET specific surface area (SSA), pore volume and pore diameter of 56 m^2^/g, 0.066 cm^3^/g and 3.1 nm, correspondingly. FE-SEM study was used to look at the surface morphology and size of particles of produced NiCuCoO materials (Fig. 1c,d). The developed samples have nanoparticles with uniform surface morphology and an average particle size of 35 nm.

**Figure 2:**
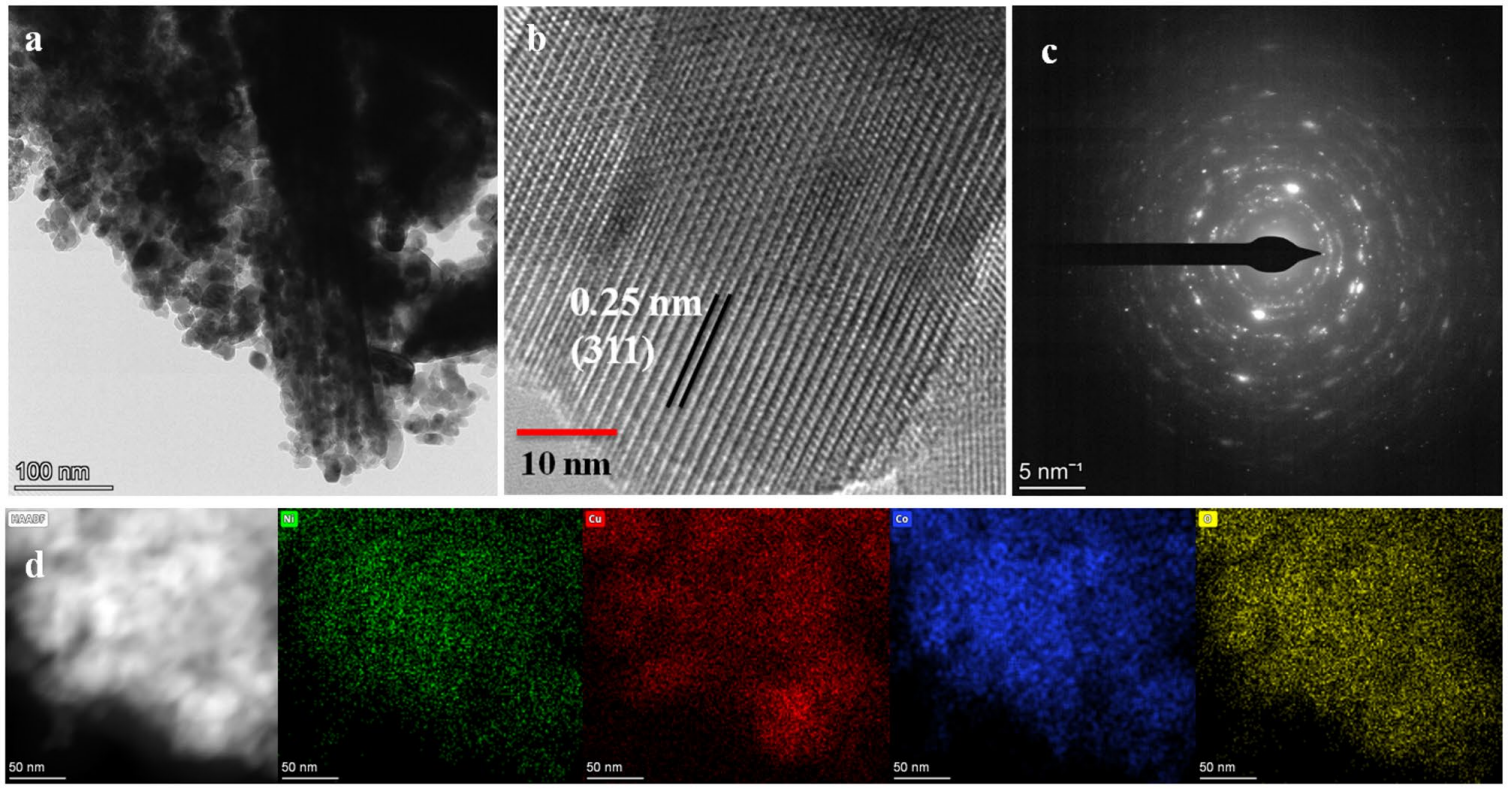
NiCuCoO NPs (**a**) TEM image, (**b**) HRTEM image (**c**) SAED pattern and (**d**) STEM and corresponding elemental plot.

To further identify every intricate structure of NiCuCoO NPs, TEM was used. From the image Fig. 2a, it is clear seen that NiCuCoO nanoparticles consist of homogenous dispersion of ultra-fine nanoparticles. HR-TEM image confirms a distance between planes d = 0.25 nm are dependable with the (311) lattice plan are proposing it has a fine crystal clear formation, which is reliable with XRD measurements (Fig. 2b). Single NiCuCoO NPs selective-area electron diffraction (SAED) patterns (insert in Fig. 2c) showed clearly defined diffraction rings, suggesting that the materials are polycrystalline. Additionally, Fig. 2d showed EDX elemental mapping images of a single NiCuCoO NPs. The homogeneous allocation of Ni (green), Cu (red), Co (blue), and O (yellow) element across every entire NiCuCoO NPs suggests that NiCuCoO successfully formed into nanoparticles-like structures.

### Supercapacitor performance

**Figure 3:**
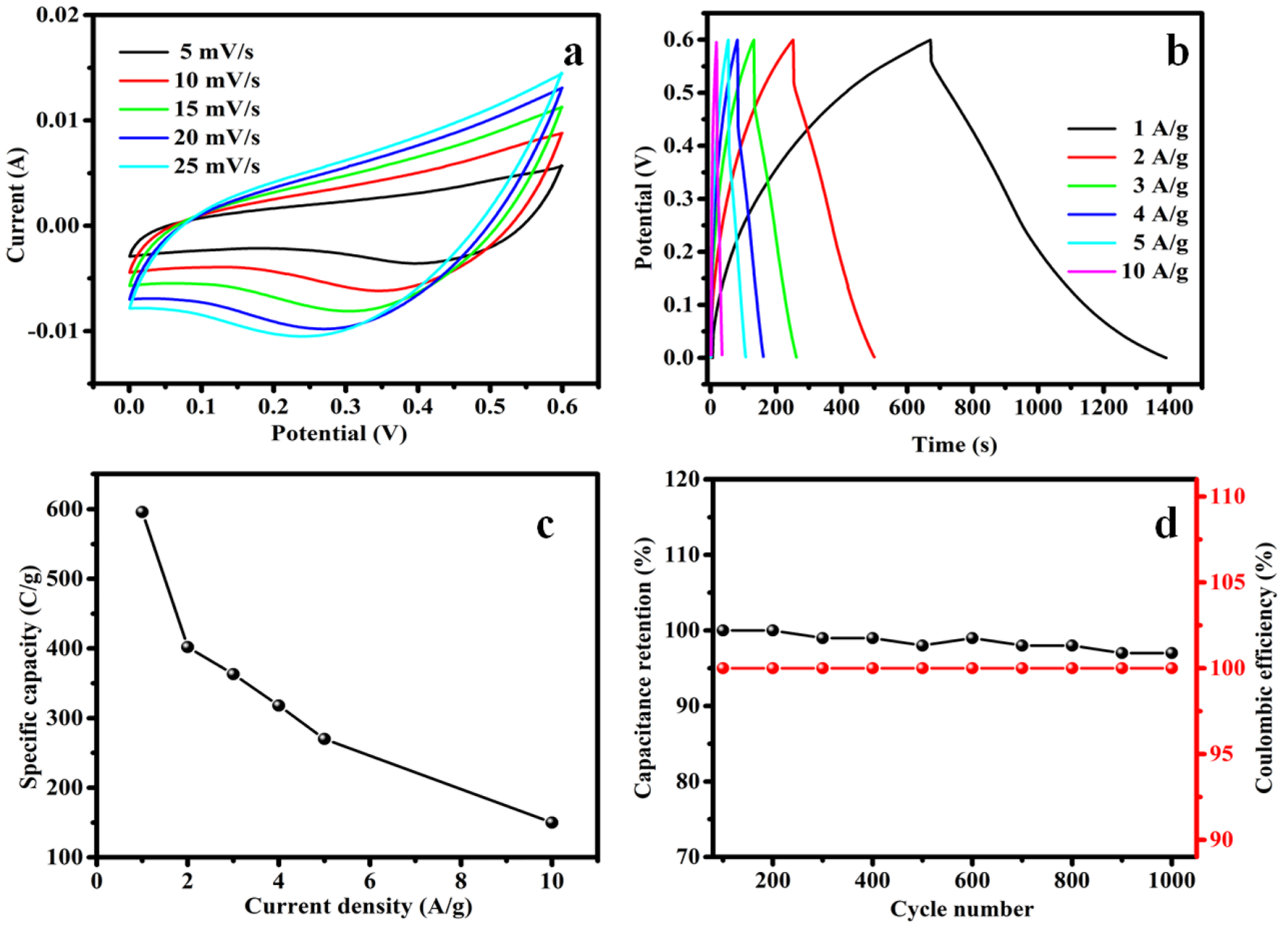
(**a**) CV profile of NiCuCoO NPs at a range of scan rate, (**b**) GCD profile of NiCuCoO NPs at a range of current density, (**c**) Specified capacity of NiCuCoO as a role of density of current and (**d**) cyclic strength and coulombic effectiveness of NiCuCoO NPs electrode materials since a purpose of sequence information.

Every typical CV curve generated by the NiCuCoO electrode is depicted in Fig. 3a at diverse scan rate ranges of 5, 10, 15, 20, and 25 mV s^−1^. Each CV curve showed two distinct redox peaks, implying that the reversible faradic reaction MO/MOOH (M indicates Ni or Co) was the primary driving force behind the electrochemical performance of the NiCuCoO electrode during anode and cathode sweeping. The oxidation as well as reduction peaks increased linearly towards positive and negative potentials with an increase in scan rate, indicating the electrode's polarization impact during the redox reaction. Furthermore, despite growing the sweep rate as 5 to 25 mV s^−1^ (a 40-fold increase in the scan rate), the outline of the CV profile still exhibited battery-type behavior, indicating the electrode material's ultrahigh rate ability and outstanding electrochemical reversibility of the redox reaction. The NiCuCoO electrode's GCD profiles are depicted in Fig. 3b for a potential choice of 0 to 0.6 V and at a variety of current densities that series as 1 to 10 A g^−1^. The GCD curve additionally explained battery-type behavior resulting plateau during the charge/discharge that confirm the faradic reactions, corresponding to the findings from the CV curve. The specific capacity of the material used for electrodes is shown since a purpose of current densities in Fig. 3c. The specified capacitance values get decreases while current density gets increases. The estimated specific capacitances of the NiCuCoO NPs as of the GCD curve are 596, 402, 363, 308, 270 and 150 C g^−1^ at density of current is 1, 2, 3, 4, 5 and 10 A g^−1^. The cycle reliability of the electrodes, as demonstrated in Fig. 3d, was tested up to 1000 cycles at an elevated density of current of 10 A g^−1^. After 1000 sequence, 99% (NiCuCoO) of the initial individual capacitance was retained. This high stability indicates that an extremely reversible surface redox reaction occurred among the electrolyte with the electrode. 100% of Coulombic efficiency for NiCuCoO electrodes after 1000 sequence at density of current 10 A g^−1^, this clearly confirms the electrochemical strength of the prepared electrodes.

**Figure 4:**
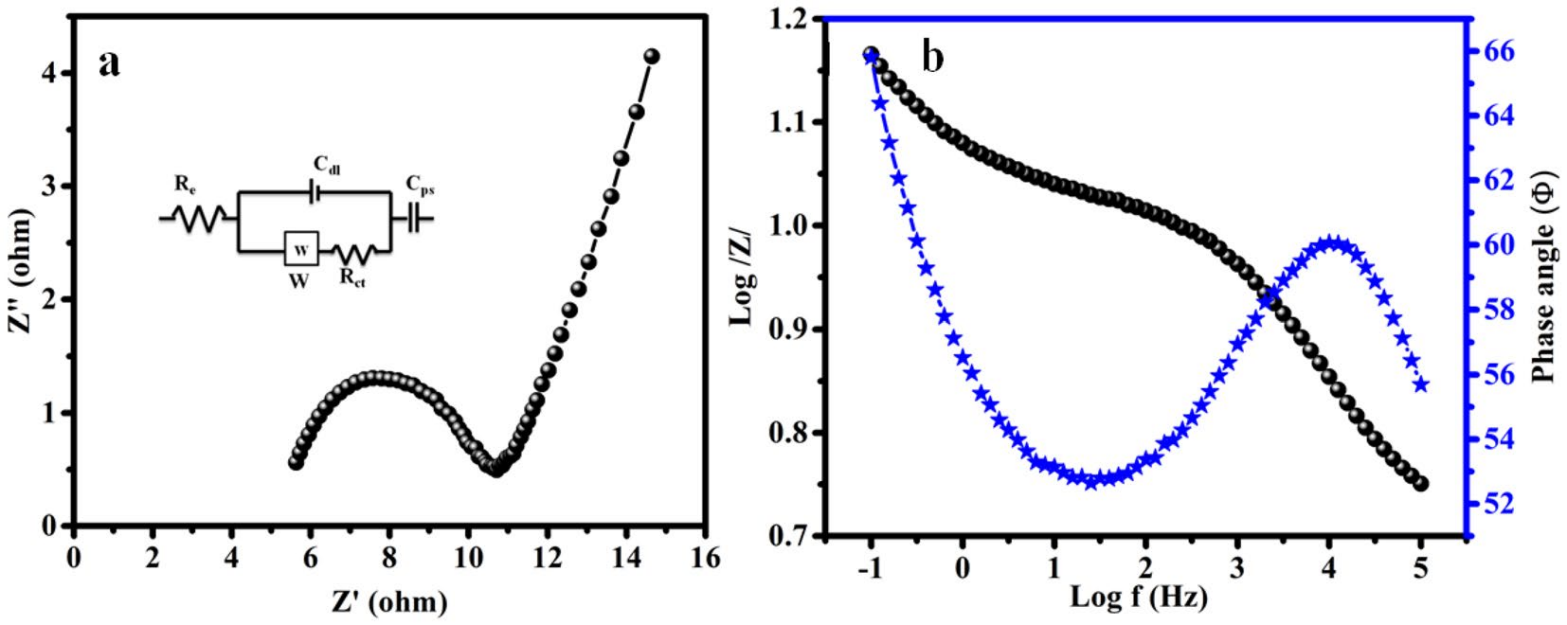
(**a**) Nyquist plot (inset) best fitted equivalent circuit (**b**) Bode plot of phase angle and log |z| *vs* frequency of the NiCuCoO electrode recorded in 100 kHz to 0.1 Hz frequency range.

Impedance analysis is used to evaluate the electrochemical rate behaviour of the NiCuCoO electrode at frequencies ranging from 100 kHz to 0.1 Hz, with amplitude of 10 mV. The Nyquist plot indicates the solution resistance, phase angle, and capacitive behaviour of electrode materials. Figure 4a displays the Nyquist plot for the NiCuCoO electrode. The diagram depicts a small semicircle representing the electrolyte resistance (R_s_ = 5.6Ω) and charge-transfer resistance (R_ct_ = 4.8Ω). The Warburg Impedance (W = 0.02) associated with semi-infinite linear diffusion (C_1_ = 2 F) is caused by the pseudocapacitance or porous electrode. The lower region shows an increase in frequency and imaginary impedance, indicating capacitive behaviour from double layer charging-discharging. Figure 4b confirms the Bode plot of the NiCuCoO electrode. The graph explains that the frequency (f_0_) matches Z_im_ peak value in the high frequency region, comparable to supercapacitors using aqueous electrolytes. The period of relaxation constant τ_0_ was found to be approximately 0.25 s using the equation τ_0_ = 1/f_0_. At 0.1 Hz, the NiCuCoO electrode had a phase angle of approximately 60° and a shallower slope due to pseudo capacitive behaviour. NiCuCoO nanoparticles reduce electrolyte resistance and facilitate K + ion de-intercalation and intercalation due to its short diffusion distance^31,32^.

**Figure 5:**
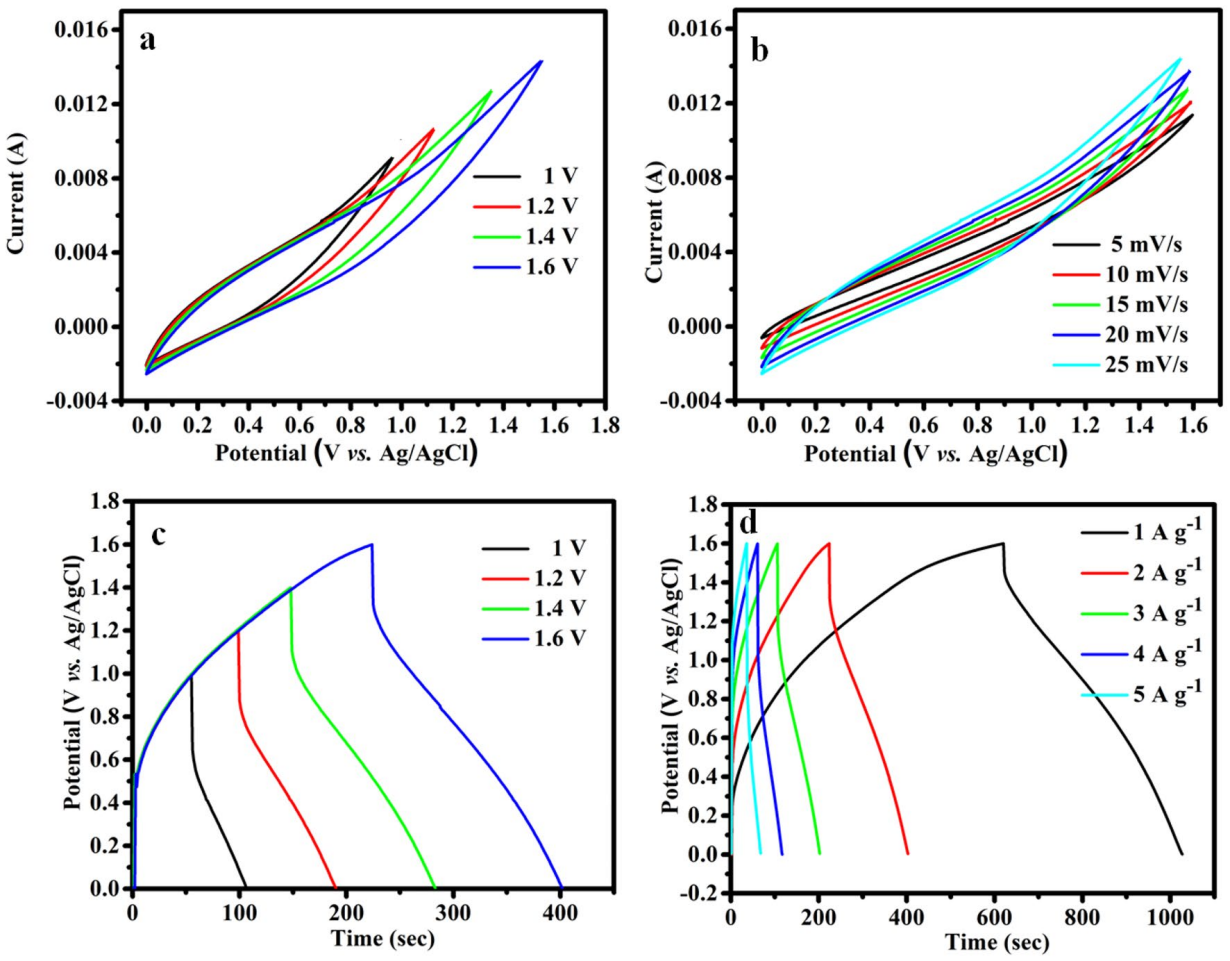
(**a**) CV profile for NiCuCoO //AC at various running potential at the sweep rate of 10 mV s^-1^, (**b**) CV curve in 1.6 V at various sweep rate, (**c**) GCD curve for ASC at various running potential at the density of current 1 A g^−1^, (**d**) GCD curve for ASC at various density of current at cell potential of 1.6 V.

Figure 5a represents the CV profile of as fabricated at diverse voltage windows as 1.0 to 1.6 V at a sweep rate of 25 mV s^-1^. Figure 5b at a cell voltage of 1.6 V present the CV profiles of the mixture NiCuCoO //AC ASC at different sweep rates variety as 5 to 25 mV s^−1^. Additionally, next to a density of current 1 A g^−1^, every GCD shape maintains a nearly shapely form even when the potential windows are raised as of 1.0 to 16 V. The CV profile sustains its shape flat at an elevated sweep rate, indicating high electrochemical convenience and resulting in good rate performance. These phenomena show that the electrochemical performance of the NiCuCoO //AC ASC device as it may be investigated using the potential window of 0–1.6 V (Fig. 5c). The CV curve's as-obtained results indicated that the gathered ASC device had pseudocapacitance belongings. Figure 5d demonstrates every ASC GCD profile at 1.6 V cell voltages with diverse densities of current variety as of 1 to 5 A g^−1^.

**Figure 6:**
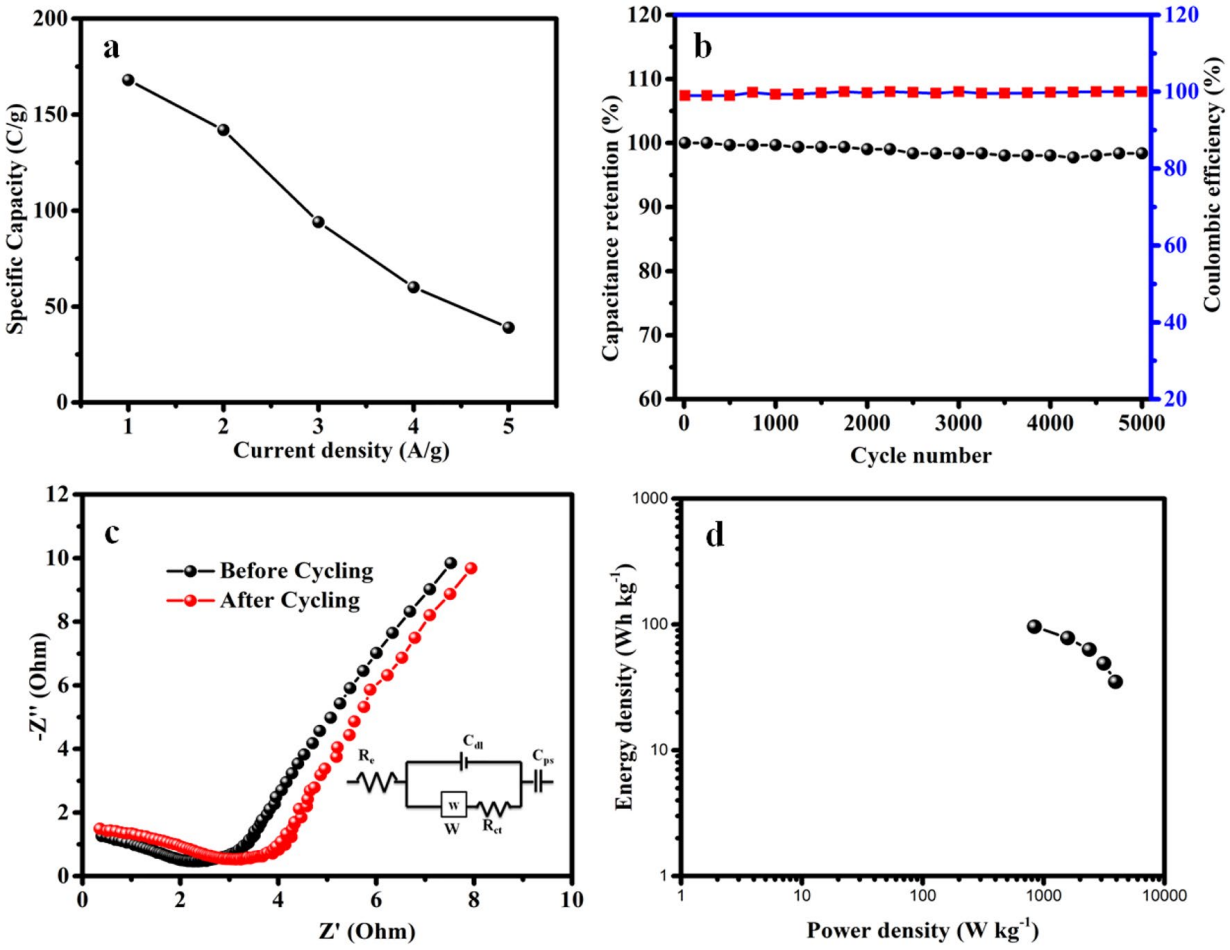
(**a**) specified capacitance obtained as a purpose density of current, (**b**) capacitance retaining and Coulombic effectiveness of assembled ASC because a purpose of sequence amount, (**c**) Nyquist plot of NiCuCoO //AC ASC and (**d**) ragone design between energy and power density.

The specific capacitance of NiCuCoO //AC ASC at different current densities are obtained based on GCD curve and result are presented in Fig. 6a. Conventional capacitors store charge proportional to the voltage applied, unlike batteries that use an electrolyte to isolate redox reactions between species with different potentials. To compare with the carbon electrode, convert its constant capacitance into C g^−1^ by calculating the width of the potential window during cycling. This allows for direct comparisons of total charge stored at each electrode^33–35^. The specific capacity gets decreases from 168, 142, 94, 60 and 39 C g^−1^ while current densities get increasing from 1 to 5 Ag^−1^. The energy efficiency of a supercapacitor can be calculated by dividing the discharge and charge curve areas, which should be close to 100% in our case the energy efficiency is 105% due to some parasitic reactions in water oxidation. Figure 6b shows that 95% of capacitance retention with 99% of columbic efficiency for all 5000 cycles. The EIS technique was used to assess the surface resistance of the materials used for electrodes at the electrode and electrolyte interfaces. The Nyquist plot of NiCuCoO NPs material for electrodes fit in a suitable equivalent circuit model is shown in Fig. 6c. Solution resistivity (R_s_), charge-transfer resistivity (R_ct_), double layer capacitance (C_dl_), Warburg impedance (W) or diffusion resistance, and faradic capacitance (C_F_) are all components of the equivalent circuit. From the fitting analysis, the NiCuCoO NPs electrode exhibits R_s_ values of 1.4 Ω, R_ct_ values of 2.7 Ω, still behind 5000 sequences; R_ct_ rises somewhat to 3.4 Ω. This slight increase in R_ct_ might be brought on by the electrolytes small solidifying or desiccation, which also had an impact on the capacitance value throughout the cycling route. Additionally, the presence of a tangent line in the Nyquist curve in the low frequency range indicates that ASC has a lower Warburg impedance (a signal of a mild ionic diffusion route), which led to the superior electrochemical routine of NiCuCoO. The results implied a superior electrical conductivity and capacitive character. The Ragone plot of the fabricated NiCuCoO //AC ASC device reveals an energy 96 Wh kg^−1^ through a power density 841 W kg^−1^ at a density of current 1 A g^−1^. When the current density improved to 5 A g^−1^, the fabricated ASC device carried a high density of power is 3937 W kg^−1^ and hold on to the density of energy is 45 Wh kg^−1^ are illustrated in Fig. 6d. The above noticed energy and power density is extensively superior than the reported values are tabulated in Table 1.

**Table 1:** Performance comparison of the proposed material with earlier reported works.

Electrode materials	Specified capacities	Energy density (Wh kg^-1^)	Capacitance retention (%)	Ref
ZnNiAlCoO	892 C g^−1^	72.4	82	^30^
MnNiCoO	638 F g^−1^	68.3	63.3	^36^
MnCoFeO_4_	675 F g^−1^	18	83	^37^
CoNiZnO	1172 F g^−1^	84.2	78	^38^
ZnNiCoO	2082 F g^−1^	37.89	88	^39^
CoNiMnO	612 F g^−1^	–	78	^40^
NiCuCoO	596 C g^−1^	96	95	This work

## Conclusion

In summary, we have successfully synthesized NiCuCoO NPs through hydrothermal method. The prepared NiCuCoO electrode delivers the pseudocapacitance performance with the specified capability of 596 C g^−1^ at the density of current 1 A g^−1^ and the retaining capacities of 99% upto 1000 sequences. More extensively, an asymmetric supercapacitor found on the NiCuCoO NPs as a positive electrode and activated carbon as a negative electrode demonstrate an elevated energy and power density of 96 Wh kg^−1^ and 841 W kg^−1^ with excellent stability of 95% and coulomic efficiency of 99% even after 5000 cycles. This result reveals the potential applications of ternary metal oxides for energy storage purpose.

## Data Availability

All data generated or analysed during this study are included in this published article.
